# Colostrum Replacement and Serum IgG Concentrations in Beef Calves Delivered by Elective Cesarean Section

**DOI:** 10.3390/vetsci11060258

**Published:** 2024-06-06

**Authors:** Manuel F. Chamorro, Miguel Saucedo, Lisa Gamsjaeger, Emily J. Reppert, Matt Miesner, Thomas Passler

**Affiliations:** 1 Department of Clinical Sciences, College of Veterinary Medicine, Auburn University, Auburn, AL 36849, USA; mzs0277@auburn.edu (M.S.); passlth@auburn.edu (T.P.); 2 Department of Clinical Sciences, College of Veterinary Medicine, North Carolina State University, Raleigh, NC 27606, USA; lgamsjaeger@ncsu.edu; 3 Department of Clinical Sciences, College of Veterinary Medicine, Kansas State University, Manhattan, KS 66506, USA; erepper@vet.k-state.edu (E.J.R.); mmiesner@vet.k-state.edu (M.M.)

**Keywords:** C-section, calf, IgG, colostrum, replacement

## Abstract

**Simple Summary:**

Inadequate consumption of maternal colostrum and lack of sufficient transfer of immunoglobulin G (IgG) to the circulation increases the risk of disease and death for beef calves. Assisted calving and cesarean section (C-section) are important risk factors for the inability of beef calves to stand, nurse colostrum from their dam, and acquire passive immunity. Colostrum replacement recommendations for beef calves, and especially for those delivered by C-section, are unavailable. The objective of this study was to determine whether or not administration of a colostrum replacement product alone or in combination with consumption of maternal colostrum would increase the serum IgG concentrations of beef calves delivered by elective C-section, compared to C-section-born calves not supplemented with colostrum. An elective C-section was performed in 32 pregnant beef cows and first-calf heifers. Immediately after delivery, newborn calves were randomly assigned to one of three different treatment groups. Group A calves (*n* = 7) were fed one packet of a commercial colostrum replacer (CR) product providing 60 g of IgG within 30 min of life. A second packet of the same CR was fed at 6 h of life. Group B calves (*n* = 13) were fed the same CR at the same frequency as group A; however, these calves were reunited with their dams after the second CR feeding to allow additional nursing of maternal colostrum. Group C calves (*n* = 12) were united with their dams immediately after surgery without colostrum intervention. Serum IgG levels at 48 h of life were greater in calves from group C and in calves born to multiparous cows. Based on the results of this study, neither colostrum replacement nor supplementation result in higher serum IgG concentrations in beef calves delivered by elective C-section compared with natural nursing.

**Abstract:**

Assistance during calving and cesarean section (C-section) are important risk factors for the failure of transfer of passive immunity (FTPI) in beef calves, which increases the risk of morbidity and mortality in beef calves during the preweaning period. Colostrum replacement recommendations for beef calves, and especially for those delivered by C-section, are unavailable. The objective of this study was to determine whether or not colostrum replacement or supplementation with a commercially available product could increase serum IgG concentrations in beef calves delivered by elective C-section, compared to beef calves that nursed colostrum naturally. An elective C-section was performed in 32 pregnant beef cows and first-calf heifers. Immediately after delivery, newborn calves were randomly assigned to one of three different treatment groups. Group A calves (*n* = 7) were fed one packet of a commercial colostrum replacer (CR) product providing 60 g of IgG within 30 min of life. A second packet of the same CR was fed at 6 h of life. Group B calves (*n* = 13) were fed the same CR at the same frequency as group A; however, these calves were reunited with their dams after the second CR feeding to allow additional nursing of maternal colostrum. Group C calves (*n* = 12) were united with their dams immediately after surgery without colostrum intervention. Serum IgG levels at 48 h of life were greater in group C calves and in calves born to multiparous cows. Based on the results of this study, neither colostrum replacement nor supplementation result in higher serum IgG concentrations in beef calves delivered by elective C-section compared with natural nursing.

## 1. Introduction

Beef-calf survival and health are important sustainability indicators of cow–calf production systems worldwide [1]. These indicators are especially important for beef seedstock producers, who make considerable investments in the genetic merit of their replacement stock. Failure of transfer of passive immunity (FTPI) due to the inadequate absorption of immunoglobulins from maternal colostrum is the most important risk factor for beef-calf morbidity and mortality [2,3,4,5]. Beef calves between 1 and 7 days of age with serum IgG levels < 10 g/L are considered to have FTPI, while calves with serum IgG levels between 10 and 24 g/L are considered to have inadequate transfer of passive immunity (ITPI) [5,6]. Results from multiple research studies demonstrated the negative impact of FTPI and ITPI on beef-calf health and performance prior to weaning [2,3,5,7]. Although several cow and calf factors have been associated with an increased risk of FTPI and/or ITPI in beef calves, dystocia (calving assistance including cesarean section) and lack of calf vigor (absence of a strong suckle reflex at birth) were most frequently demonstrated to be important contributors to low serum IgG levels, poor health, and increased treatment risk prior to weaning [8,9,10]. In conventional North American cow–calf farms, approximately 6% of cows need assistance during calving, and approximately 2.6–3.6% of dystocia cows require a cesarean section (C-section) for calf delivery [11,12]. This surgery is commonly performed in cattle admitted to referral or veterinary teaching hospitals, where elective C-sections are performed in pregnant cattle carrying offspring of exceptional genetic value to avoid complications of dystocia. Additionally, with the recent growth in using beef-bull semen in dairy herds (“beef on dairy”) to produce calves for the beef industry could lead to an increased practice of elective C-sections in dairy cattle to prevent the negative consequences of dystocia in cows and calves. However, increased occurrences of hypoxemia and a greater risk of failure to adapt to extrauterine-life have been reported in calves delivered by elective C-section [13,14,15]. In some instances, dams used as embryo recipients may not produce enough high-quality colostrum, thus increasing the risk of FTPI or ITPI in their calves. Therefore, producers and veterinarians often administer colostrum replacement products to calves delivered by C-section to improve calf survival; however, evidence-based guidelines for colostrum supplementation and replacement in beef calves are currently unavailable. Recommendations for colostrum replacement in dairy calves call for the administration of a high volume (~4 L) of high-quality (>22% Brix) maternal colostrum or colostrum replacer powder (300 g of IgG) within the first 2–4 h of birth [16,17]. These recommendations may be impractical for beef calves for several reasons, including lack of complete separation of the dam, excessive volume in relation to calf body weight, potential disruption of nursing behavior due to gastrointestinal distention, and colic [18,19,20]. The overarching goal of colostrum supplementation and/or replacement in beef calves should be to minimize the negative impact of FTPI and ITPI while avoiding disruption of normal nursing behavior [18,21]. Therefore, the main objective of this study was to determine if administration of a commercially available colostrum replacer alone or in combination with maternal colostrum increases the serum IgG concentrations of beef calves delivered by elective C-section in a veterinary teaching hospital. We hypothesized that a combination of colostrum replacement and nursing of maternal colostrum would result in greater serum IgG concentrations at 48 h of life compared with either strategy alone.

## 2. Materials and Methods

### 2.1. Experimental Design and Sample Collection

Thirty-two multisource, crossbreed, multiparous beef cows [20] and first-calf heifers [12] were enrolled in this study from February 2017 through March 2018. Cows and heifers were synchronized with a modified 7-d CIDR-synch protocol and artificially inseminated (AI) as previously described [22]. On the same day, a 3-year-old Hereford clean-up bull with a satisfactory breeding soundness examination was added to the pasture with inseminated cattle to ensure greater rates of pregnancy. Pregnancy in the 32 animals was confirmed by rectal palpation and ultrasound examination 25, 45, 90, 240, and 280 d following AI. At approximately 280 d of gestation, all cows were moved into individual stalls heated to approximately 21 °C at the Veterinary Health Center, College of Veterinary Medicine, Kansas State University. Individual stalls were equipped with a head gate and a swing gate that allowed adequate restrain of the cow and exposure of the left flank. Following individual physical examination, parturition was induced in all dams with a combination of 20 mg of dexamethasone (Dexamethasone^®^ 2 mg/mL, Agrilabs, Schuyler, NE, USA) and 25 mg of dinoprost (Lutalyse^®^, Zoetis, Parsipanny, NJ, USA), administered intramuscularly as previously described [23]. Two days following induction of parturition, elective left-flank C-sections were performed in all cows as previously described [24]. Immediately after delivery, newborn calves were placed in sternal recumbency and airway patency was established by removing fluid from the nasopharynx with a 60 mL bulb syringe (Blowout Medical, Cottonwood Heights, UT, USA). Towel rubbing was performed on the thorax and limbs of calves to stimulate respiration. Once calves were stable and breathing normally, they were weighed on an electric scale (Livestock Platform Scale^®^ Brecknell, Fairmont, MN, USA) and moved in front of their dams to allow licking by their dams and promote bonding. 

Calves were randomly assigned to 3 different treatment groups using a random number generator (Microsoft Excel^®^, Microsoft, Redmond, WA, USA). Group A (*n* = 7) was fed two packets of a commercial colostrum replacer (CR) product (Calf Choice Total Gold^®^, Saskatoon Colostrum Company, Saskatoon, SK, Canada). Each packet was mixed according to manufacturer directions to a final volume of 800 mL, containing 60 g of immunoglobulin G (IgG). The first packet was fed within 30 min of delivery, and a second packet was fed at 6 h of life. Calves in group A remained separated from their dams for 48 h to prevent natural nursing of maternal colostrum. At 12 h after the second feeding of CR, group A calves were fed 1.5 L of a commercial milk replacer (28% crude protein, 20% crude fat Cow’s match^®^, Land O’ Lakes, Arden Hills, MN, USA) mixed according to label directions (283.5 g of milk replacer powder in 2 L of water) twice daily until reunion with their dams at 48 h of age. Group B (*n* = 13) was fed the same CR product at the same quantity and frequency as group A; however, calves were reunited with their dams immediately after the second feeding of CR to allow the additional nursing of maternal colostrum. Colostrum replacement treatments were administered to all calves in groups A and B using an esophageal feeder (JorVet Oral Calf Feeder, Jorgensen Laboratories, Inc., Loveland, CO, USA). Group C calves (*n* = 12) were placed in the stall with their dams immediately after surgery was completed to allow natural nursing of maternal colostrum without intervention. 

Flunixin Meglumine (Banamine^®^, Merck Animal Health, Rahway, NJ, USA), at 1.1 mg/kg of body weight (BW) intravenously every 24 h for up to 3 days, was administered to all cows to control pain and inflammation post-surgery. Two doses of Oxytetracycline LA 200 (Bio-Mycin^®^, Boehringer Ingelheim Animal Health, Duluth, GA, USA), at 20 mg/kg BW subcutaneously 48 h apart, were administered prophylactically to all cows to prevent inadvertent post-surgical infection.

Blood samples were collected from all calves at 48 h of life to determine levels of IgG in the serum. All calves were examined twice daily during the first 48 h of life by a veterinarian (M.S.) blinded to treatment allocation. The presence of clinical signs of disease such as fever (rectal temperature > 39.7° F), lethargy, and diarrhea was evaluated and recorded. The apparent efficiency of absorption (AEA) of IgG was calculated for group A with the following formula: serum IgG concentration at 48 h of life (g/L) × plasma volume (L) ÷ total IgG intake (g), as previously described [25]. Following blood sample collection, all cow–calf pairs were moved into a single dry pen for post-operative monitoring. The Kansas State University Institutional Animal Care and Use Committee (IACUC) PRN # 2016-3693 reviewed and approved all animal protocols. 

### 2.2. Determination of Total Serum IgG by Radial Immunodiffusion

Serum samples were diluted (1:4) and assayed for IgG concentration by single radial immunodiffusion as previously described [25]. Single radial immunodiffusion plates were prepared from 2% agarose containing 2.5% Rabbit anti-bovine IgG-1 (H an L chains; Bio-Rad, Hercules, CA, USA) in phosphate-buffered saline (VeltivexTM^®^ sodium chloride injection solution 0.9%, Dechra Veterinary Products; Overland Park, KS, USA). Standard curves (1.06–8.5 g/L IgG) were calculated using duplicate samples of a bovine IgG serum calibrator (Midlands Bio Products Corp., Boone, IA, USA). All samples were tested in triplicate and incubated in a humid atmosphere at 25 °C for 18–24 h. The diameter of the rings were measured with a computer-assisted plate reader (The Binding Site, Birmingham, UK), and an average of the 3 readings was used to assign IgG concentration in each sample.

### 2.3. Statistical Analysis

Statistical analyses were performed using a commercially available software package (JMP^®^ 16.0.0, SAS Institute Inc., Cary, NC, USA). Descriptive statistics, including mean, median, standard deviation, minimum, and maximum were calculated. Data were assessed for normality by visual inspection of frequency distributions and by the Shapiro–Wilk test, indicating that the main outcome variables of interest, serum IgG concentration at 48 h of life and body weight at birth, were normally distributed. Statistical tests were performed to test the null hypotheses that serum IgG concentrations were not different between groups (one-way ANOVA with group as the main treatment factor, followed by post-hoc comparison using Tukey’s HSD), between male and female calves (Student’s *t*-test), or between calves that nursed maternal colostrum (groups B and C) born to first-calf heifers or multiparous dams (Student’s *t*-test). Furthermore, the proportion of calves with either adequate, inadequate, or failure of transfer of passive immunity was compared between groups using the Fisher’s exact test. For all statistical tests, a value of *p* < 0.05 was considered significant.

## 3. Results

### Demographic Data and Serum IgG Levels

All cows were good surgery candidates (apparently healthy) based on physical examination before the induction of parturition. Group A contained 7 multiparous cows and no first-calf heifers, group B contained 7 multiparous cows and 6 first-calf heifers, and group C contained 6 multiparous cows and 6 first-calf heifers. All C-section surgeries were uneventfully completed (skin incision to skin suture) in approximately 90 min, and all calves (17 heifers and 15 bulls) were delivered without complications. The distribution of calf sex between treatment groups was 4 bulls and 3 heifers in group A, 6 bulls and 7 heifers in group B, and 5 bulls and 7 heifers in group C. All calves remained healthy and without signs of clinical disease during the first 48 h of life. A significant effect of sex on serum IgG at 48 h of life was not detected (*p* > 0.05). The overall mean individual birth weight (kg) ± SEM was 29.9 ± 0.52 kg. The mean birth weight ± SEM for calves in groups A, B, and C were 30.1 ± 0.89 kg, 30.3 ± 0.88 kg, and 29.4 ± 0.92 kg, respectively, which was not significantly different (*p* = 0.74). 

**Figure 1:**
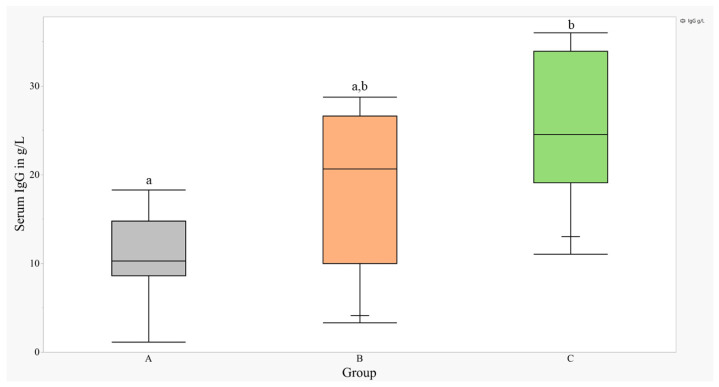
Mean serum IgG ± SEM levels at 48 h of life in beef calves delivered by elective C-section. Within 6 h of life calves were fed either 2 doses of a commercial colostrum replacer providing 120 g of IgG (A), 2 doses of a commercial colostrum replacer providing 120 g of IgG and additional natural nursing of maternal colostrum (B), or maternal colostrum through natural nursing only (C). Distinct letters (small caps a, b) above each box plot represent significant (*p* < 0.05) differences between treatment groups (A, B, C).

At 48 h of life, a statistically greater (*p* < 0.01) mean serum IgG ± SEM was detected in group C (25.5 ± 2.3 g/L) compared with group A (10.5 ± 4.3 g/L); however, the mean serum IgG ± SEM in group B (18.4 ± 2.2 g/L) was not significantly different compared with that of groups A and C (Figure 1). The overall prevalence of FTPI (Serum IgG < 10 g/L) and ITPI (serum IgG 10–24 g/L) was 18.7% (6/32) and 50% (16/32), respectively. Only 31.5% (10/32) of all calves achieved adequate transfer of passive immunity (ATPI) based on the beef-calf standard of serum IgG > 24 g/L [6]. A greater proportion of calves from group A had either FTPI or ITPI compared with groups B and C in the same categories, and a greater proportion of calves from group C had ATPI compared with groups A and B in the same category (Table 1); however, these differences were not statistically significant (Table 1, *p* = 0.06). In calves from groups B and C, a statistically greater (*p* < 0.01) mean serum IgG ± SEM was detected in calves born to multiparous cows compared with calves born to first-calf heifers (27 ± 1.7 g/L vs. 16.1 ± 2.4 g/L). The median IgG AEA from group A calves was 21.9%. The AEA was not calculated for groups B and C because the total IgG mass consumed from natural nursing of maternal colostrum was unknown. 

## 4. Discussion

The prevalence of failure of FTPI (serum IgG < 10 g/L) and ITPI (serum IgG > 10 < 24 g/L) in beef calves from groups A, B, and C was 43%, 23%, and 0%, and 57%, 46%, and 50%, respectively. The prevalence of FTPI and ITPI of groups A and B was greater compared with a 0% FTPI and 6.6% ITPI reported in a previous study in which a colostrum replacement (CR) intervention of a similar volume and IgG concentration was administered to beef calves within 60 min of birth [18]. The prevalence of FTPI in group C (0%) was similar to the 5% FTPI rate previously reported in a population of 420 beef calves that nursed maternal colostrum naturally from their dams [6]. In contrast, the prevalence of ITPI in group C calves (50%) was greater compared to the ITPI rates of 18% and 37% previously reported for different populations of beef calves that nursed maternal colostrum naturally from their dams [2,6]. The mean serum IgG at 48 h of life was lower in calves that received CR (groups A and B) compared with calves that solely nursed maternal colostrum from their dams post-surgery (group C). While we expected that serum IgG from calves in group B would be greatest compared with other groups, our results demonstrate that colostrum supplementation was not beneficial in this case. These results are consistent with previous studies in beef calves in which any form of colostrum intervention (administration of maternal colostrum or a CR by bottle or tube) resulted in lower serum IgG and increased the odds of FTPI and ITPI when compared to calves that nursed their dams [2,6]. However, other factors including total mass of IgG and feeding times could have played a role in the lower-than-expected serum IgG levels and AEA observed in calves from group A. It is likely that the total mass of IgG fed to calves from group A (120 g) was too low to provide adequate serum IgG levels. When compared with IgG concentrations normally present in maternal colostrum from beef cows (>100 g/L), and manufacturer’s feeding recommendations for the utilized CR (total of 180 g of IgG), the administered 120 g of IgG appeared to have been too low for adequate serum IgG levels at 48 h [26]. Additionally, feeding the second dose of CR at 6 h of life, rather than sooner, may have decreased the efficiency of IgG absorption in group A. An additional treatment group receiving frozen maternal colostrum was not included in this study because our study population represented beef calves from North American cow–calf beef farms in which the practice of collecting and freezing maternal colostrum is very rare, and therefore adding a group with frozen maternal colostrum would not have represented typical management of these operations. 

Serum IgG at 48 h was significantly lower in calves born to first-calf heifers compared with calves born to multiparous cows. This is consistent with results from previous studies in beef calves [2,5]. Compared to multiparous cows, first-calf beef heifers may have lower mammary gland development, production of colostrum, and serum IgG levels in their calves [19]. Additionally, the results from some studies demonstrated lower volume, IgG concentration, and spectrum of colostral antibodies in maternal colostrum from first-calf beef heifers compared with multiparous cows [5,6,27]. The results from another study suggested that first-calf Belgian Blue heifers undergoing elective C-sections licked their newborn calves less and demonstrated reduced suckling behavior (mothering ability) compared with Belgian Blue cows undergoing elective C-section [28]. All these factors could help explain the lower serum IgG levels observed in calves born to first-calf heifers in our study. In contrast, the results from one study demonstrated a mean of 30 g/L serum IgG in neonatal calves born to first-calf beef heifers [29]. Other factors such as esophageal irritation by tubing and satiation could also have negatively affected nursing behavior and IgG absorption in calves from group B [30], and measuring the concentration of IgG in maternal colostrum would have been ideal to determine the role of maternal colostrum quality on serum IgG levels, FTPI, and ITPI in groups B and C.

Elective cesarean section (C-section) in cattle provides a better prognosis for calf survival compared with an emergency C-section [31]; however, the results from one study suggested that prolonged duration of elective C-sections (>111 min) in beef cows resulted in maternal colostrum of lower quality [27]. Although the duration of elective C-sections in our study was approximately 90 min, longer surgical procedures could have affected colostrum quality and IgG absorption in calves from groups B and C. Additionally, the induction of parturition before 270 days of gestation increases the risk of lung immaturity and respiratory distress syndrome (RDS) in newborn calves [32,33]. In our study, parturition was induced after 270 days of gestation in all cows and none of the calves developed clinical signs of RDS; however, mild effects of induction of parturition on lung maturity could have affected the extrauterine adaptation of calves and reduced IgG absorption from colostrum. One study demonstrated an increased risk of hypoxemia in calves delivered by elective C-section compared with unassisted vaginally-delivered calves [15]. The authors of that study proposed that lack of pelvic transit and thoracic compression in calves delivered by elective C-section affected oxygenation post-delivery. Hypoxemia because of elective C-sections could have disrupted the cerebral responses associated with adaptation (i.e., suckle reflex) and reduced IgG absorption in the small intestine in calves from our study [13,34]. Unfortunately, we did not evaluate vigor parameters (i.e., suckle reflex, mucous membranes color, tongue withdrawal, etc.), arterial oxygen pressure/saturation, or nursing behavior in calves from our study. This information would have been important to determine the role of these factors on the low levels of serum IgG observed in some calves across different treatment groups.

Colostrum supplementation (giving a colostrum replacer product in addition to natural nursing of maternal colostrum) and complete replacement of colostrum in beef calves is challenging because the goal is to minimize the risk of FTPI while at the same time promoting natural nursing and maternal bonding. The results from a previous study demonstrated that feeding beef calves a CR protocol of medium volume (1.4 L) and moderate IgG concentration (70 g/L) resulted in reduction in the time at which calves stood up and nursed from their dam compared with protocols of higher IgG concentration or volume [18]. This CR protocol is similar to the CR protocol used in our study. However, in this population of beef calves delivered by elective C-section, CR of medium volume (1.6 L) and moderate IgG concentration (120 g) alone or in combination with natural nursing did not provide advantages in increasing serum IgG levels, or in preventing FTPI and ITPI, when compared with natural nursing of maternal colostrum alone.

Several limitations could have influenced the results of our study, including the lack of assessment of mammary gland conformation and colostrum quality, the lack of evaluation of calf vigor and nursing behavior, and the administration of a lower dose of IgG than that recommended by the CR manufacturer. Evaluating mammary gland conformation, maternal colostrum quality, calf vigor, and nursing behavior could have provided insights into their role on the lower-than-expected serum IgG levels observed in group B and greater-than-expected proportion of FTPI and ITPI observed in groups B and C. Poor mammary gland conformation (e.g., bottled teats) and lower quality of maternal colostrum (IgG concentration < 100 g/L) are important risk factors for FTPI in beef calves [19,26]. Differences in the number of cows with poor udder conformation in groups B and C, and in the quality of maternal colostrum from cows in the same groups, could have affected the levels of serum IgG observed in their calves. In a similar manner, differences in the presence and/or strength of the suckle reflex and other vigor parameters as well as in nursing behavior in calves from groups B and C could have provided insights into the differences in serum IgG levels observed in these groups. Unfortunately, this information was not collected in our study, and inferences on its effect in the results of our study would be speculative.

The label of the CR product used in this study states that “for complete replacement of maternal colostrum, 3 packets (180 g of IgG) must be fed to calves within 8 h of life”. It is possible that using three instead of two packets of the CR could have resulted in greater serum IgG levels in group A; however, it is unknown if this could have improved serum IgG levels in group B. The decision to use two instead of three packets of the product was made because we wanted to evaluate a CR strategy of medium volume that would result in greater natural nursing.

## 5. Conclusions

Complete colostrum replacement or colostrum supplementation with 120 g of IgG within the first 6 h of life of apparently healthy beef calves delivered by elective C-section was not superior to allowing natural nursing of maternal colostrum following surgery on preventing failure in the transfer of passive immunity (FTPI) in the population of calves from this study. The information on colostrum replacement practices for beef calves delivered by elective C-section generated in this study can be used to educate producers, veterinarians, and veterinary students and therefore improve overall beef-calf health and the sustainability of cattle production systems. Additional research on colostrum replacement strategies for beef calves delivered by C-section analyzing the effect of factors such as mammary gland conformation, maternal colostrum quality, calf-vigor, and nursing behavior on long-term health and performance outcomes are necessary in order to refine management strategies in this population of animals.

## Figures and Tables

**Table 1:** Proportion of failure of transfer of passive immunity (FTPI), inadequate transfer of passive immunity (ITPI), and adequate transfer of passive immunity (API) based on serum IgG levels at 48 h of life among beef calves from groups A, B, and C delivered by elective C-section. Identical letters (small caps a) indicate lack of detection of significant differences (*p* > 0.05) between groups in each category.

Group	FTPI (IgG < 10 g/L)	ITPI (IgG ≥ 10 < 24 g/L)	API (IgG ≥ 24 g/L)
A	3/7 (43%) ^a^	4/7 (57%) ^a^	0/7 (0%) ^a^
B	3/13 (23%) ^a^	6/13 (46%) ^a^	4/13 (31%) ^a^
C	0/12 (0%) ^a^	6/12 (50%) ^a^	6/12 (50%) ^a^

## Data Availability

The data presented in this study are available on request from the corresponding author.
